# Exogenous spermidine improved drought tolerance in *Ilex verticillata* seedlings

**DOI:** 10.3389/fpls.2023.1065208

**Published:** 2023-01-20

**Authors:** Xiaoting Xie, Yujie Gu, Weili Wang, Farhat Abbas, Sini Qin, Siyi Fu, Jiaqi Mei, Jiayan Wang, Dexuan Ma, Guangchao Wen, Ying Yang, Anket Sharma, Xiaofei Wang, Daoliang Yan, Bingsong Zheng, Yi He, Huwei Yuan

**Affiliations:** ^1^ State Key Laboratory of Subtropical Silviculture, Zhejiang Agricultural and Forestry University, Hangzhou, China; ^2^ Zhejiang Provincial Key Laboratory of Forest Aromatic Plants-based Healthcare Functions, Zhejiang Agricultural and Forestry University, Hangzhou, China

**Keywords:** drought, *Ilex verticillata*, physiological responses, spermidine, stress

## Abstract

Winterberry (*Ilex verticillata* (L.) A. Gray) is a recently introduced ornamental tree species in China that has not been closely investigated for its drought resistance. In this study, we used two-year-old cuttings from *I. verticillata* (L.) A. Gray and two representative varieties derived from it, *I. verticillata* ‘Oosterwijk’ and *I. verticillata* ‘Jim Dandy’, as materials to investigate how this plant responds to drought stress and whether exogenous spermidine (SPD) can alleviate the negative effects caused by drought stress. The results showed that as the degree of drought stress increased, the leaves of winterberry seedlings became chlorotic, and their edges became dry. Similarly, the relative water content, specific leaf weight, chlorophyll content, leaf nitrogen content, net photosynthetic rate, stomatal conductance and transpiration rate were significantly reduced, whereas the content of malondialdehyde continuously increased with the degree of drought stress. The activities of superoxide dismutase, peroxidase, and catalase increased under moderate drought stress and then decreased under severe drought stress. The levels of soluble sugar and abscisic acid continued to increase, while those of auxin and gibberellic acid decreased. When compared with individual drought stress, an increase in the amount of external SPD clearly alleviated the effect of drought stress on winterberry seedlings. The combined phenotypes and physiological indices of the winterberry leaves under drought stress conditions revealed that the drought resistance of the native species was significantly higher than its two varieties. This finding serves as an important theoretical foundation for the popularization and application of *I. verticillata* (L.) A. Gray and the two varieties.

## 1 Introduction

With recent population growth and a developing global economy, increasing water scarcity is a major reason for the global expansion of arid and semiarid zones (ASZ) (Dubois et al., 2018; Liang et al., 2018; Huang et al., 2019). Drought stress has become one of the most important abiotic factors, which causes massive crop loses (Lou et al., 2020; Zhao et al., 2020). Drought stress has an impact on plant metabolism by inhibiting cell elongation, which reduces the leaf area available for photosynthesis. With increasing abiotic stresses, the leaf water potential decreases, and the net photosynthesis rate (Pn) also continuously decreases until the leaf curls, yellows, and wilts; it can no longer support photosynthesis, and the plant dies (Hill et al., 2013; Mohammadi et al., 2020). Plants adjust their morphological and physiological characteristics in response to drought during the early stages of drought stress to maintain growth and development. Plants reduce cell membrane damage by participating in the defensive capability of antioxidants to increase antioxidant enzyme activities, and plants improve the leaf water potential by osmoregulation under drought conditions to retain the leaf water content and delay the decrease in photosynthesis. Several studies have found that plants adapt to drought stress by regulating plant hormones to maintain various antioxidant enzyme activities *in vivo*. It is critical to research plant drought resistance, as well as improve drought resistance and water use efficiency in plants (Wa et al., 2014; Bai et al., 2015).

Polyamines have a wide range of phytohormonal regulation effects in higher plants, including the delay of growth hormones in plants that cause aging. While aging-promoting hormones, such as ethylene, can inhibit the synthesis of polyamines, these compounds directly affect plant growth and development by regulating plant hormones (Guo et al., 2018). Polyamines have been shown to resist biological and abiotic stress in higher plants and play an important role in plant growth and development, morphological formation, aging, and dormancy, and they are receiving increasing attention in the study of plant resistance (Igarashi and Kashiwagi, 2000; Gill and Tuteja, 2010; Minocha et al., 2010; Chen et al., 2017; Chen et al., 2019). Plant tolerance was reduced by inhibitors of polyamine biosynthesis, but plant tolerance was restored by concurrent treatment with exogenous polyamines (He et al., 2002). This supports the concept that polyamines play an important role in plant environmental tolerance. It has also been reported that polyamines can act as signaling molecules in stress signal transduction and help to build mechanisms to resist stress (Kasukabe et al., 2004). Spermidine (SPD) has been linked to stress resistance and acts as a direct stress protector in studies of polyamine stress resistance. SPD has also been shown to regulate plant hormones, stabilize cell membranes and antioxidant systems, and act as an osmoregulatory substance during responses to plant stress.

SPD regulates the alteration of osmoregulatory substances caused by drought stress and participates in plant drought resistance as an osmoregulatory substance. McNeil et al. (1999) demonstrated that SS and soluble nitrogen compounds could significantly accumulate in stressed plants under drought stress, and SPD could alleviate drought by reducing osmoregulatory substances. Previous research has shown that exogenous SPD can effectively alleviate the effects of salt stress on winterberry (*Ilex verticillata*) (Tian, 2017).

*Ilex verticillata* (L.) A. Gray, a dioecious shrub that is native to the northeastern United States, has the characteristics of falling leaves and a long fruiting period in the autumn and winter, which makes it an excellent ornamental tree species for cut branches (Chen et al., 2015; Yang et al., 2016). These shrubs are popular for container gardens and landscaping owing to their bright red color. *I. verticillata* is known to adapt to grow in a humid environment. However, its potential to grow in more drought stricken environments has been less well studied, whether SPD is suitable for alleviating the negative effects of drought stress on *I. verticillata* seedlings is never studied, and the levels of drought tolerance among different *I. verticillata* varieties has less been compared. In this study, the growth status of *I. verticillata* cuttings under different levels of drought stress, the effects of exogenous SPD on the tolerance of *I. verticillata* seedlings to drought stress, as well as the differences in drought tolerance among different varieties of *I. verticillata*, were investigated in terms of morphological and physiological characteristics. The results from this study will provide a more theoretical foundation and practical exploration for the expansion of its application in arid and semiarid regions.

## 2 Materials and methods

### 2.1 Plant materials

Plant materials, including cuttings of the *I*. *verticillata* (L.) A. Gray, *I*. *verticillata* ‘Oosterwijk,’ *I*. *verticillata* ‘Jim Dandy’, were planted in the greenhouse of Zhejiang A&F University (Hangzhou, China) (N 30°23’, E 119°72’) under the following conditions: a day/night (12 h/12 h) cycle at 35°C/25°C (day/night) and 65% relative humidity. After 3 months of cultivation inside the greenhouse, eight treatment groups were established using the weighing method described below in **Table 1**. For different levels of drought stress treatments, after calculating the field holding capacity of the culture medium, the soil moisture contents were maintained at 25%-30% (severe drought), 40%-45% (moderate drought), 55%-60% (slight drought), or 80%-85% (control) of the field holding capacity, respectively. Five seedlings were used in each treatment group of a certain verity. In this study, the leaves of the plant material were sprayed with 0.5 mmol·L^-1^ SPD every other day from 0 d to the extent that the leaf surface and leaf back were wet but not dripping, while the control was sprayed with the same conditions of water. The leaves were harvested at 0, 7, and 30 days after the treatments. Three biological replicates were performed for each time point. Leaves with the same location and similar size and shape (with the length of about 10 cm and the width of about 5 cm, oval) were collected as samples in different treatment groups for determination of different parameters. In addition, 10 leaves were removed and photographed on the day of sampling for each treatment.

**Table 1:** Treatment groups.

Number	Groups	Treatment
CK	Control	Control watered without SPD and keep soil moisture content at 80-85% of the field holding capacity
D1	Mild drought stress	Keep 55%-60% of the field holding capacity
D2	Moderate drought stress	Keep 40%-45% of the field holding capacity
D3	Severe drought stress	Keep 25%-30% of the field holding capacity
CK+SPD	Control + SPD	Control watered with SPD and keep soil moisture content at 80-85% of the field holding capacity
D1+SPD	Mild drought stress + SPD	Keep 55%-60% of the field holding capacity with SPD
D2+SPD	Moderate drought stress + SPD	Keep 40%-45% of the field holding capacity with SPD
D3+SPD	Severe drought stress + SPD	Keep 25%-30% of the field holding capacity with SPD

### 2.2 Determination of the relative leaf water content and specific leaf weight

Fresh leaves were initially washed with moist blotting paper to determine the relative leaf water content. Secondly, the fresh weight (Wf) of leaves was measured. Third, the saturated weight of leaves (Wt) was measured after several times of 12 h periods of water absorption until a constant weight had been reached. Fourth, the leaves were dried at 80°C until a constant weight and the dry weight (Wd) of leaves was measured. Finally, the relative leaf water content (%) was calculated as (Wf-Wd)/(Wt-Wd)×100%. The mean was regarded as the relative leaf water content of the corresponding group.

The specific leaf weight (SLW) was determined by selecting, washing, drying, and photographing mature leaves in the middle of cuttings. Image J software (NIH, Bethesda, MD, USA) was used to calculate the leaf area (S). Next, fresh leaves were blanched at 105°C for 30 min and then dried at 80°C until a constant weight to determine the Wd. The SLW was calculated as Wd/S as described by Chen et al. (2017).

### 2.3 Measurement of the photosynthetic parameters

The net photosynthetic rate (Pn), stomatal conductance (Gs), transpiration rate (Tr) and leaf internal CO_2_ (Ci) of leaves were measured using a Li-6400 (LI-COR, Lincoln, NE, USA) according to the manufacturer’s instructions. The data were collected between 8:00 and 11:30 during the treatment period. Three to five round healthy mature leaves (grown normally for 3 months) with a similar area (about 10 cm in length and 5 cm in width) were selected for study. The leaf chamber temperature, atmospheric CO_2_ concentration, flow rate, and photosynthetically active radiation were all kept constant at 30°C, 400 cm^3^·m^-3^, 500 μmol·s^-1^, and 1,800 μmol·m^-2^·s^-1^, respectively, during the treatment period.

### 2.4 Determination of the chlorophyll fluorescence parameters

The upper and middle mature leaves of the plants were chosen and acclimated in the dark for more than 25 min before a rapid light curve was measured with a PAM2500 fluorometer (Walz, Effeltrich, Germany) as described by Tian et al. (2016). During determination, the activation light intensity was set as 1,000 μmol·m^-2^·s^-1^. A total of 12 light intensity gradients, including 980, 788, 622, 477, 366, 274, 201, 144, 104, 67, 9 and 5 μmol·m^-2^·s^-1^, were used during the fast light curve. The instrument directly provides the data for all the chlorophyll fluorescence parameters.

### 2.5 Determination of the contents of chlorophyll and nitrogen

A plant nutrient meter was used to determine the chlorophyll and nitrogen content of the leaves (TYS-3N; Zhejiang Top Instruments Co., Ltd., Hangzhou, China). Leaves with uniform growth conditions and similar size were chosen from each group. The instrument was preheated for 2-5 min and then set at zero three times. Secondly, leaves were put on the instrument for determination. The data from each leaf were read 3-5 times, and there were three replicates for each group.

### 2.6 Determination of the contents of soluble sugar and malondialdehyde, as well as the activities of superoxide dismutase, peroxidase, and catalase

The contents of soluble sugar (SS) was determined using the anthrone colorimetry method (Yemm and Willis, 1954). First, a standard curve was created: (1) different volumes of 100 μg·mL^-1^ glucose (from 0 mL to 1.0 mL with 0.2 mL interval) and up to 1.0 mL of distilled water were added to six test tubes; (2) A volume of 5 mL anthrone reagent was added into each of the tubes; (3) After rapid shaking, the mixture of each tube was heated in boiling water for 10 min; (4) After cooling, the content of glucose in each tube was measured by spectrophotometry at a wavelength of 620 nm; (5) A standard curve was created. Secondly, the soluble sugar in the samples was extracted as follows: (1) 0.1 g fresh leaves were cut into pieces and added to a tube; (2) After 15 mL of distilled water had been added, the tube was heated in boiling water for 20 min; (3) After cooling, the sample was filtered, put in a 100 mL volumetric flask, and diluted with distilled water to volume. Finally, 5 mL anthrone reagent was mixed with 1 mL of extraction solution and used for measurement.

To determine the content of malondialdehyde (MDA) and the activities of superoxide dismutase (SOD), peroxidase (POD), and catalase (CAT), 0.5 g of frozen leaves were ground into powder in liquid nitrogen in a pre-chilled pestle and mortar. After adding 5 mL 0.05 mol·L^-1^ phosphate buffer (pH 7.8), the mixture was centrifuged at 6000 rpm for 30 min. The supernatant was regarded as solution to determine the different parameters. The MDA content was measured using the thiobarbituric acid method as described by Chen et al. (2017). The activity of SOD was measured using an SOD assay kit. The CAT activity was determined using the UV absorption assay (Guo et al., 2016). The POD activity was measured using the guaiacol method as described by Ying et al. (2013). The activities of SOD, POD, and CAT were determined as described by Chen et al. (2017).

### 2.7 Measurement of endogenous hormone content

Each sample was ground to powder in in liquid nitrogen in a pre-chilled mortar. Secondly, a 10% tissue homogenate was made by adding 0.9 mL of 0.01 mol·L^-1^ phosphate buffer solution (pH 7.2-7.4) into 0.1 g of sample. The tissue homogenate was then centrifuged at 4000 rpm for 15 min at 4°C. The supernatant was used for plant hormone extraction by different kits. The concentrations of plant hormones auxin (IAA), gibberellin (GA), and abscisic acid (ABA) were measured using ELISA kits following the manufacturer’s instructions (MB-3372, MB 3379, Hufeng Chemical Co., Shanghai, China). Finally, 10 μL of supernatant was added to the microplate reader for data collection at a wavelength of 450 nm (Chen et al., 2017).

### 2.8 Statistical analysis

SPSS 22 (IBM, Inc., Armonk, NY, USA) was used to conduct the statistical analyses. The data were analyzed using a one-way analysis of variance (ANOVA) followed by a Duncan’s test at a 5% significance interval, and each experiment was repeated three times.

## 3 Results

### 3.1 Exogenous SPD alleviated the damage of leaves under drought stress

**Figure 1:**
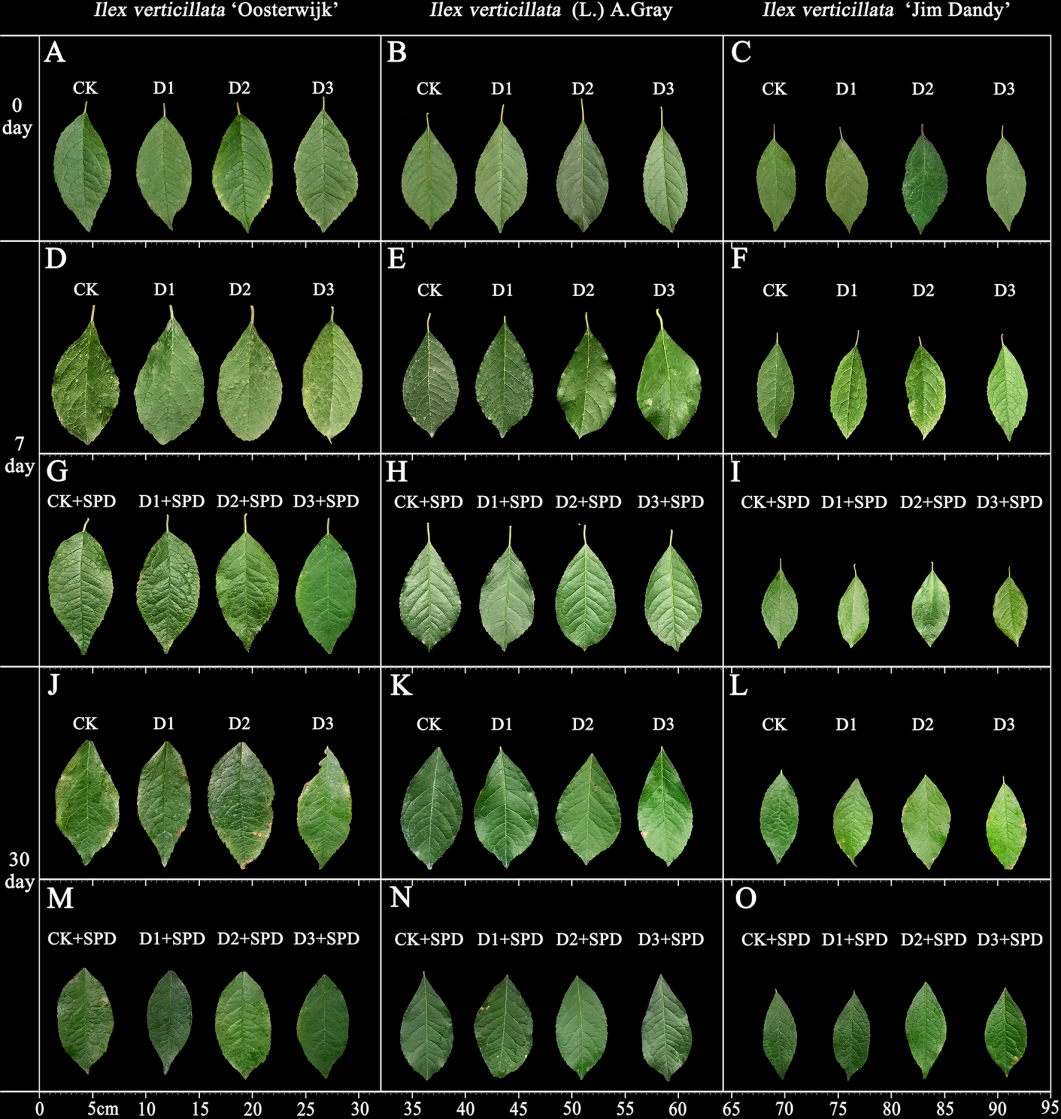
The phenotypic changes of *Ilex verticillata* leaves under drought and spermidine treatments. **(A, D, G, J, M)** represent the phenotypes of *I. verticillata* ‘Oosterwijk’ leaves. **(B, E, H, K, N)** represent the phenotypes of *I. verticillata* (L.) A. Gray leaves. **(C, F, I, L, O)** represent the phenotypes of *I. verticillata* ‘Jim Dandy’ leaves. A-C represent the phenotypes of the leaves under different treatments on day 0. **(D-I)** represent the phenotypes of the leaves under different treatments on day 7. **(J-O)** represent the phenotype of the leaves under different treatments on day 30. CK, control; CK+SPD, control with spermidine; D1, mild drought; D1+SPD, mild drought with spermidine; D2, moderate drought; D2+SPD, moderate drought with spermidine; D3, severe drought; D3+SPD, severe drought with spermidine.

The phenotypic changes in the leaves of *I. verticillata* seedlings under different levels of drought stress treatment were different for both unsprayed and sprayed SPD treatments. After 30 days of treatment with varying levels of drought stress, the surface of all the leaves gradually became lighter with curling and brown, and even drying of the leaf edges. Moreover, as the drought stress increased, the number and area of dry patches increased (**Figures 1J–L**). Under severe drought stress, *I*. *verticillata* ‘Oosterwijk’ even displayed defective leaf edges. In contrast, the leaf color of the SPD-treated group improved significantly after spraying (**Figures 1M–O**). In terms of leaf morphology, leaves of the native species were the least affected by drought stress compared with the other two varieties, indicating that the native species is more resistant to drought stress than the two varieties artificially selected for aesthetic purposes.

We measured the relative water content (RWC) of the leaves to assess their water status and retention capacity. The RWC of *I. verticillata* leaves decreased gradually with increasing duration and degree of drought stress in all the three different varieties (**Table 2**). The RWC of leaves treated with SPD under varying degrees of drought stress decreased in comparison to the control group but increased significantly in comparison to those not sprayed with SPD. Among the three varieties used, the RWC of the native species increased the most significantly by more than 6 percent after treatment with SPD under severe drought stress.

**Table 2:** Changes in the relative water content of *Ilex verticillata* leaves under drought and spermidine treatments.

Varieties	Groups	Relative water content (%)		
		0d	7d	30d
*I. verticillata* ‘Oosterwijk’	CK	94.94 ± 0.32a	94.42 ± 1.45a	94.42 ± 1.45a
	CK + SPD	94.42 ± 1.45a	94.94 ± 0.32a	94.94 ± 0.32a
	D1	94.34 ± 0.72a	83.50 ± 0.50d	77.55 ± 1.96d
	D1 + SPD	94.41 ± 1.06a	91.85 ± 0.40b	83.66 ± 2.55b
	D2	94.42 ± 1.45a	81.69 ± 0.60e	76.71 ± 2.31d
	D2 + SPD	94.94 ± 0.32a	85.32 ± 0.83c	80.94 ± 0.40c
	D3	94.50 ± 0.91a	77.90 ± 1.47f	73.73 ± 0.70e
	D3 + SPD	94.02 ± 1.12a	79.67 ± 1.54f	78.55 ± 1.00cd
*I. verticillata* (L.) A. Gray	CK	93.50 ± 0.67a	93.50 ± 0.85a	92.09 ± 0.92a
	CK + SPD	93.50 ± 0.76a	93.50 ± 0.76a	93.50 ± 0.67a
	D1	93.50 ± 0.95a	85.11 ± 0.60d	77.98 ± 1.26e
	D1 + SPD	93.50 ± 0.85a	89.99 ± 1.33b	87.75 ± 0.73b
	D2	93.50 ± 0.67a	84.80 ± 0.92d	75.94 ± 0.74f
	D2 + SPD	93.50 ± 0.76a	89.50 ± 0.57b	86.08 ± 0.26c
	D3	93.50 ± 0.67a	81.10 ± 0.82e	74.95 ± 1.10f
	D3 + SPD	93.50 ± 0.67a	87.01 ± 0.58c	81.44 ± 1.03d
*I. verticillata* ‘Jim Dandy’	CK	92.70 ± 1.20a	91.98 ± 0.20a	91.91 ± 0.24a
	CK + SPD	92.70 ± 1.20a	92.37 ± 0.67a	92.70 ± 1.20a
	D1	92.89 ± 1.02a	80.74 ± 1.63cd	72.87 ± 1.20d
	D1 + SPD	92.89 ± 1.02a	85.39 ± 2.61b	76.27 ± 1.29b
	D2	92.70 ± 1.20a	78.52 ± 1.83de	72.65 ± 1.30d
	D2 + SPD	92.89 ± 1.02a	82.87 ± 2.71bc	75.58 ± 1.73bc
	D3	92.89 ± 1.02a	77.29 ± 1.70e	68.71 ± 1.49e
	D3 + SPD	92.89 ± 1.02a	82.54 ± 1.70bc	73.78 ± 0.94cd

CK, control; CK+SPD, control with spermidine; D1, mild drought; D1+SPD, mild drought with spermidine; D2, moderate drought; D2+SPD, moderate drought with spermidine; D3, severe drought; D3+SPD, severe drought with spermidine. Data on 0 d, 7 d and 30 d were collected for a one-way analysis of variance (ANOVA) (P < 0.05, n = 3), respectively. Multiple comparisons were conducted using Duncan’s method. Different lowercase letters after the data on the same day indicate a significant difference between different treatments, while the same lowercase letters indicate a non-significant difference.

**Figure 2:**
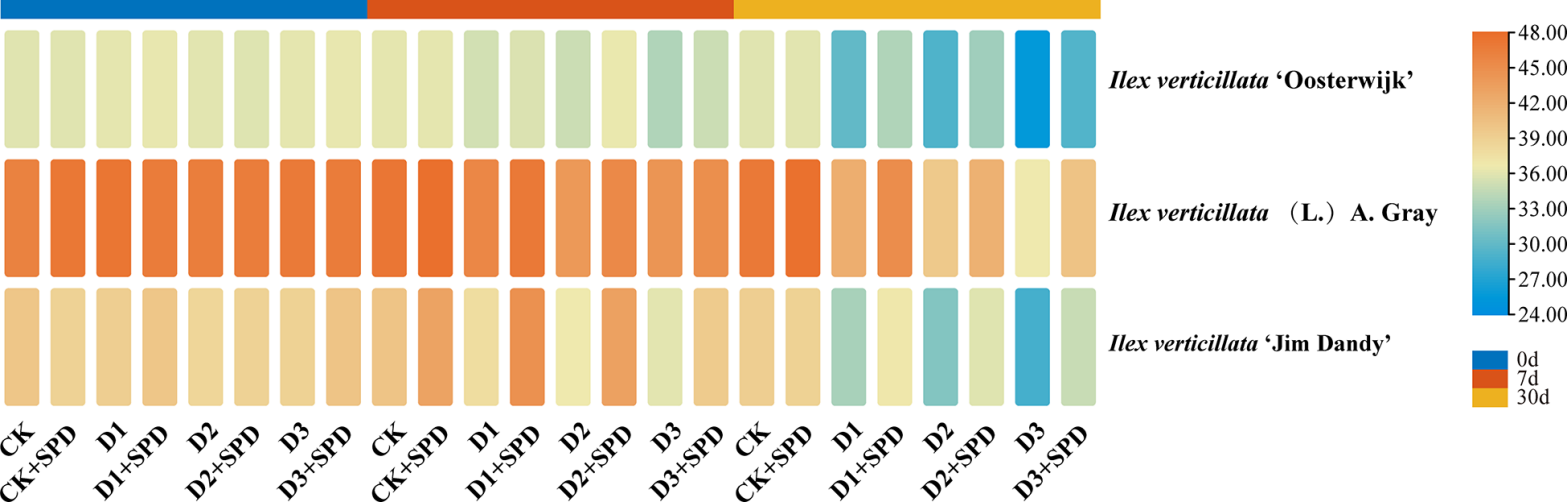
The changes of specific leaf weight in *Ilex verticillata* leaves under drought and spermidine treatment. Data were shown in a heatmap, where orange indicates high values and blue indicates low values. CK, control; CK+SPD, control with spermidine; D1, mild drought; D1+SPD, mild drought with spermidine; D2, moderate drought; D2+SPD, moderate drought with spermidine; D3, severe drought; D3+SPD, severe drought with spermidine.

The specific leaf weight (SLW) of the leaves of the three varieties gradually decreased with increasing drought stress and time (**Figure 2**). To some extent, exogenous SPD could improve the reduction in SLW of leaves caused by drought stress. Under severe drought stress for 30 days, the spraying of SPD significantly increased the SLW of *I. verticillata* leaves compared with the corresponding non-sprayed groups.

### 3.2 Exogenous SPD influenced the photosynthetic physiological indicators in drought-stressed leaves

**Figure 3:**
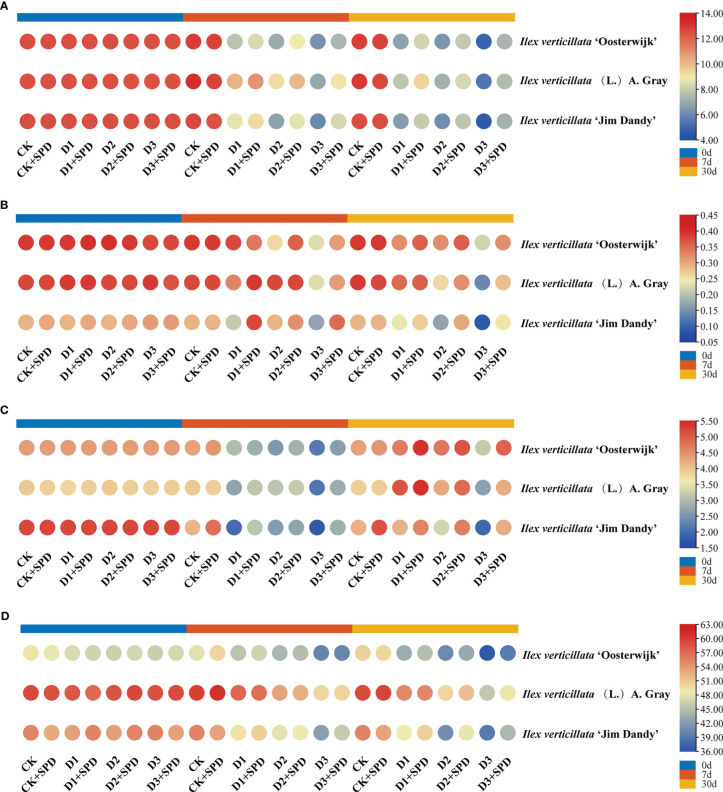
The changes of photosynthetic parameters in *Ilex verticillata* leaves under drought and spermidine treatment. **(A)** photosynthetic rate; **(B)** stomatal conductance; **(C)** transpiration rate; **(D)** chlorophyll content. Data were shown in different heatmaps, where red indicates high values and blue indicates low values. CK, control; CK+SPD, control with spermidine; D1, mild drought; D1+SPD, mild drought with spermidine; D2, moderate drought; D2+SPD, moderate drought with spermidine; D3, severe drought; D3+SPD, severe drought with spermidine.

The photosynthetic rate of leaves in the control treatment was maintained at approximately 12 μmol**·**m^-2^-s^-1^ (**Figure 3A**) with an overall trend of decreasing photosynthetic rate as the degree and duration of drought stress increased. The photosynthetic rates of leaves under various levels of drought stress were significantly reduced after exogenous SPD application compared with the control but increased significantly under severe drought stress compared with the treatment group without the application of exogenous SPD. The application of SPD significantly increased the leaves of the ‘Jim Dandy’ variety by 20.7% after 30 days of severe drought stress.

Drought stress reduced the Gs of leaves significantly more than the concurrent control (**Figure 3B**), whereas the leaves sprayed with SPD under severe drought stress increased the Gs significantly more than leaves not treated with SPD. Of these, the Gs of ‘Jim Dandy’ leaves increased significantly by 57.03% and 64.01% when the SPD was applied at 7 d of mild and severe drought stress, respectively, compared with the non-SPD treated group. The application of SPD at 30 d of moderate and severe drought stress significantly increased the Gs by 43.18% and 54.95%, respectively.

We measured the Tr of leaves to determine how much water plant leaves lose through transpiration. The findings revealed that the Tr of leaves generally decreased as drought stress increased (**Figure 3C**). In contrast, long-term exogenous SPD treatment had a mitigating effect on leaf Tr under severe drought stress.

Under drought stress, the Ci of *I. verticillata* leaves decreased compared with the control (**Supplementary Figure 1**). At 7 days of mild drought stress, the application of SPD to *I*. *verticillata* ‘Jim Dandy’ leaves increased the leaf Ci by 7.35% compared with the untreated group. The exogenous application of SPD to the leaves of ‘Oosterwijk’ after 30 d of drought stress significantly increased Ci by 7.99%. Moreover, there were no significant differences between the other treatment groups.

We measured the chlorophyll content to demonstrate the role of exogenous SPD in the efficiency of light energy uptake and use by leaves under drought stress. The chlorophyll content of ‘Jim Dandy’ leaves sprayed with SPD at 7 d of severe drought stress increased by 6.49% compared with the control that lacked SPD (**Figure 3D**). The chlorophyll content significantly increased by 13.02% and 9.53% by applying SPD at 30 d of moderate and severe drought stress, respectively, compared with no application of SPD. The chlorophyll content in leaves of the native species treated with SPD increased by 5.7% after 30 days of severe drought stress compared with the leaves that were not treated with SPD. The results of this study suggested that SPD might have a protective effect on chlorophyll in the leaves of the native species and ‘Jim Dandy’ under drought stress.

Nitrogen is an essential plant nutrient, and its abundance affects leaf size and color. Nitrogen, on the other hand, is a component of chlorophyll in plants. Thus, the amount of nitrogen in the leaves is closely related to the amount of chlorophyll. The changes in leaf nitrogen content were similar to those in the chlorophyll content, and they decreased as the time of drought stress increased (**Supplementary Figure 2**). Results indicated that exogenous SPD increased the content of nitrogen of the three varieties under drought stress.

### 3.3 Exogenous SPD influenced the chlorophyll fluorescence parameters of drought-stressed leaves

**Figure 4:**
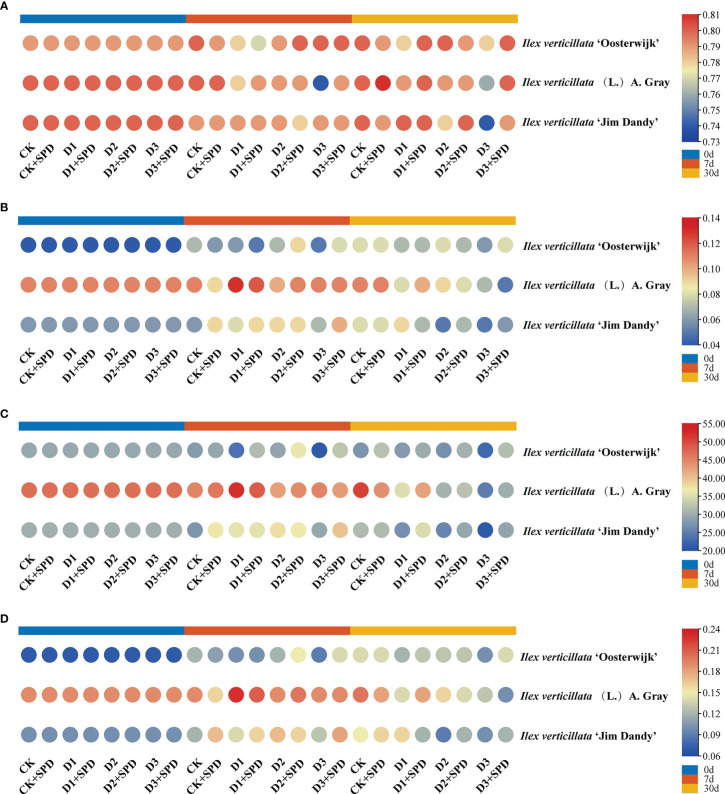
The changes of chlorophyll fluorescence parameters in *Ilex verticillata* leaves under drought and spermidine treatment. **(A)** Fv/Fm; **(B)** Y(II); **(C)** ETR; **(D)** qP. Data were shown in different heatmaps, where red indicates high values and blue indicates low values. CK, control; CK+SPD, control with spermidine; D1, mild drought; D1+SPD, mild drought with spermidine; D2, moderate drought; D2+SPD, moderate drought with spermidine; D3, severe drought; D3+SPD, severe drought with spermidine.

To reflect the degree of plant stress by adversity, we measured chlorophyll fluorescence. The maximum photochemical quantum yield of PSII is Fv/Fm, which reflects the maximum PSII light energy conversion efficiency. This is the plant’s potential maximum photosynthetic capacity. Under severe drought stress conditions, all the Fv/Fm decreased (**Figure 4A**). When exogenous SPD was sprayed on leaves after 30 days of severe drought stress, the Fv/Fm of all the three varieties increased dramatically. The most significant increase in Fv/Fm was 7.03% in the ‘Jim Dandy’ leaves compared with those without the SPD treatment. Y(II) is the actual photochemical quantum yield of PSII, which reflects the actual PSII light energy conversion efficiency. After 30 days of severe drought stress treatment, Y(II) decreased significantly compared with the corresponding control group, while exogenous SPD could alleviate the decrease in Y(II) of ‘Oosterwijk’ and ‘Jim Dandy’ but not the native species (**Figure 4B**). ETR is the relative electron transfer efficiency, which is closely related to the changes in light conditions. The changes of ETR after 30 days of severe drought stress treatment and the alleviating effect of SPD were similar to those of Fv/Fm (**Figures 4A-C**). After 30 days of severe drought stress treatment, photochemical quenching (qP) decreased significantly compared with the corresponding control group, while exogenous SPD could alleviate the decrease in qP of all the three varieties (**Figure 4D**). The non-photochemical quenching (NPQ and qN) levels remained relatively high after drought stress, and the effects of SPD treatments on NPQ and qN were not significantly different (**Supplementary Figure 3**).

### 3.4 Exogenous SPD affected the content of MDA and levels of antioxidant enzymes in drought-stressed leaves

**Figure 5:**
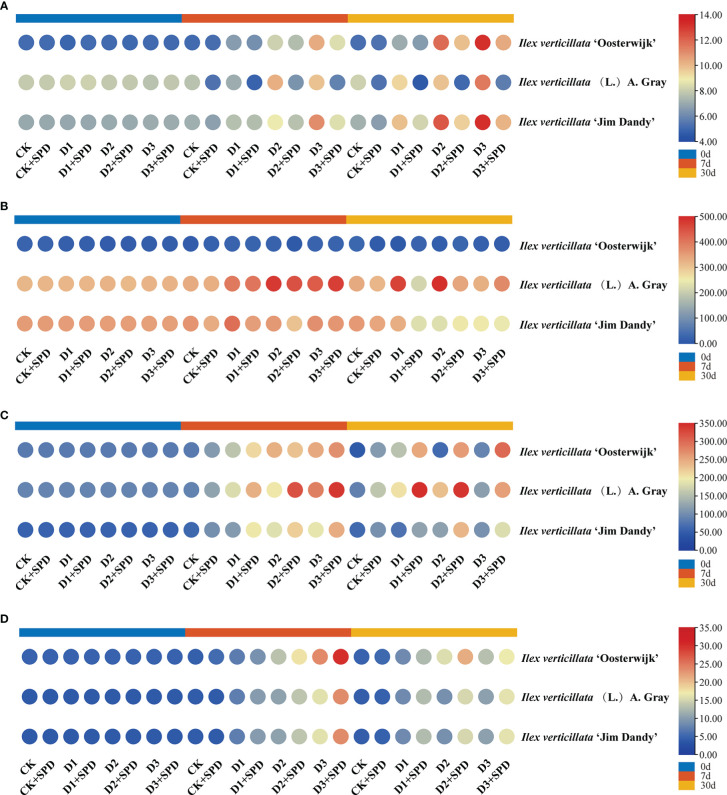
The changes in malonaldehyde content and antioxidant enzymes in *Ilex verticillata* leaves under drought and spermidine treatment. **(A)** MDA; **(B)** SOD; **(C)** POD; **(D)** CAT. Data were shown in different heatmaps, where red indicates high values and blue indicates low values. CK, control; CK+SPD, control with spermidine; D1, mild drought; D1+SPD, mild drought with spermidine; D2, moderate drought; D2+SPD, moderate drought with spermidine; D3, severe drought; D3+SPD, severe drought with spermidine. CAT, catalase; MDA, malondialdehyde; POD, peroxidase; SOD, superoxide dismutase.

The MDA content of the leaves of all the three varieties increased significantly after 30 d of drought stress when compared with the control (**Figure 5A**). The MDA content of the leaves was significantly reduced in A. Gary leaves but increased in the leaves of the two varieties after exogenous SPD treatment when compared with the control group. As time passed and the degree of drought stress increased, the leaves accumulated increasing amounts of MDA, and the MDA content decreased to varying degrees compared with those after exogenous SPD application.

Under various degrees of drought stress, the SOD activity of ‘Oosterwijk’ increased and then decreased over time. Under slight and moderate drought stress, the SOD, POD, and CAT activities of the native species continuously increased with time and followed by a decrease under severe drought stress **(** **Figures 5B-D** **)**. The SOD activity of ‘Jim Dandy’ leaves decreased over time under various degrees of drought stress; the POD activity increased followed by a decrease, and the CAT activity usually increased over time under various degrees of drought stress. Under various levels of drought stress, the activity of CAT generally increased over time.

### 3.5 Exogenous SPD influenced the drought-induced changes in SS and endogenous hormones

**Figure 6:**
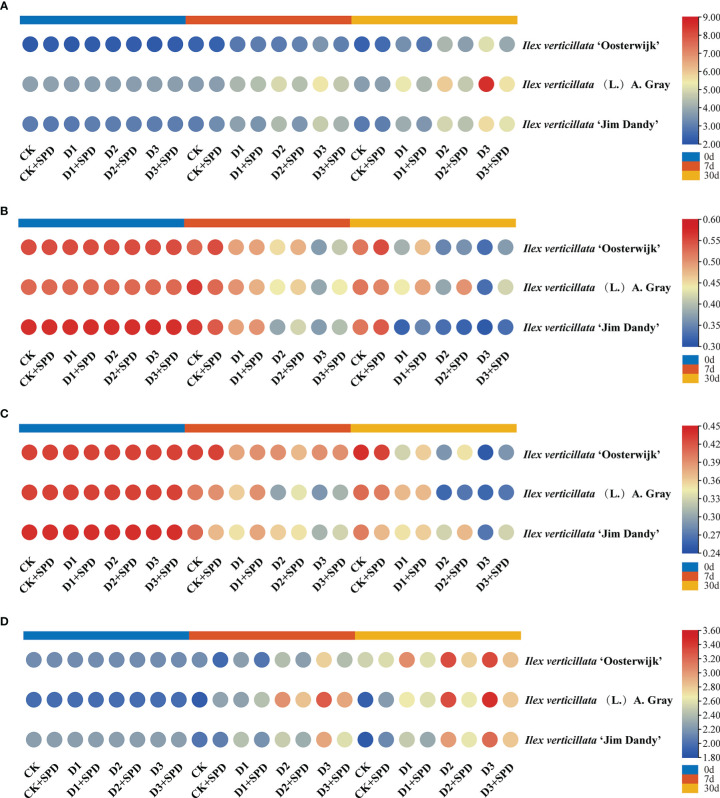
The changes of soluble sugar content and endogenous hormone contents in *Ilex verticillata* leaves under drought and spermidine treatment. **(A)** soluble sugar; **(B)** IAA; **(C)** GA; **(D)** ABA. Data were shown in different heatmaps, where red indicates high values and blue indicates low values. CK, control; CK+SPD, control with spermidine; D1, mild drought; D1+SPD, mild drought with spermidine; D2, moderate drought; D2+SPD, moderate drought with spermidine; D3, severe drought; D3+SPD, severe drought with spermidine. ABA, abscisic acid; GA, gibberellic acid; IAA, auxin.

In addition to providing energy for plant growth and development, SS are important intermediates in plant metabolism and the regulation of osmotic stress (Liu et al., 2020). The SS content of the leaves of all the three varieties accumulated gradually with time and drought, and the exogenous application of SPD affected the accumulation of SS content to varying degrees (**Figure 6A**). Under moderate and severe drought treatments for 7 and 30 days, the SS content of SDP sprayed leaves was significantly decreased compared the corresponding non-sprayed group (**Figure 6A**).

The IAA contents of ‘Oosterwijk’ and the native species leaves decreased significantly with the time delay and worsening drought (**Figure 6B**). The IAA content of the native species leaves treated with external SPD after 30 days of severe drought stress was significantly higher than those not treated with SPD.

GA is abundantly synthesized in growing seeds and fruits and is important in regulating physiological processes, such as seed germination, stem elongation, leaf growth, dormant bud germination, and flower and seed development (Dunbar-Co et al., 2009; Wang, 2013; Zhou et al., 2013). Different levels of drought stress were found to significantly reduce the GA content of the leaves compared with the control. The GA content of ‘Oosterwijk’ and ‘Jim Dandy’ leaves decreased with time and increasing drought, and exogenous SPD application played a role in the increase in leaf GA content during the late stage of drought stress. The GA content in leaves of the native species decreased significantly during the early stages of drought stress compared with the control, and exogenous SPD application significantly increased the GA content compared with the non-SPD group (**Figure 6C**).

The ABA content of the leaves of all the three varieties increased gradually over time and during drought (**Figure 6D**). The ABA content of ‘Jim Dandy’ leaves under different drought stresses after application with SPD was significantly reduced compared with the group without SPD application during the same period. Furthermore, the exogenous SPD inhibited the increase in ABA content as time and drought increased. At the onset of drought stress, the content of ABA in A. Gray leaves was significantly higher than that in the control, whereas the application of exogenous SPD significantly decreased the content of ABA compared with the non-SPD group.

## 4 Discussion

### 4.1 Exogenous SPD improves drought tolerance in plants

Drought stress is a major environmental factor that limits global plant growth and crop productivity and is one of the abiotic stresses that affects plant growth and development. It can damage plant cell membranes and nuclei, affect water content and distribution in plants, reduce plant photosynthesis, and inhibit leaf and root growth (Wang et al., 2007; Xiao et al., 2009). Plants frequently adapt to or mitigate drought stress through a series of physiological and biochemical adjustments, such as reduced plant transpiration, elimination of intracellular reactive oxygen species (ROS), and osmoregulation. Finding compounds that reduce the harmful effects of drought stress may be important from both a theoretical and practical standpoint. Numerous studies have shown that the application of exogenous SPD has been shown to increase the content of polyamines in plants, which are involved in processes, such as signal transduction, osmoregulation, and scavenging ROS (Aziz et al., 1997; Aziz et al., 1999; Liu et al., 2006; Ahmad et al., 2012). Therefore, exogenous SPD could enhance the tolerance of plants to drought stress, making SPD an important participant in the study of drought tolerance in plants. In this study, under drought stress, the leaves of *I. verticillata* curled and turned brown, and the relative water content, SLW, RWC, photosynthetic parameters, as well as IAA and GA contents significantly decreased while the content of MDA and the activities of antioxidant enzymes increased, causing negative effects on the normal growth of seedlings. In comparison, when exogenous SPD was sprayed under drought stress, the leaf morphology improved, the RWC and photosynthesis rate increased, the MDA content decreased, and the activities of antioxidant enzymes increased further. Consequently, exogenous SPD treatment mitigated the degree of damage caused by drought stress to *I. verticillata* seedlings, which was in accordance with previous studies (Abid et al., 2022).

Dryness, curling, wrinkling, and necrotic spots on the leaves are the most visible signs of the adverse effects of drought stress on the plant, while the amount of dry matter that accumulates is a direct response to the plant’s drought resistance. The water-holding capacity of plant leaves is reflected in the leaf RWC, and less drought resistance correlates with a quicker decrease in the RWC of leaves (Marshall et al., 2000). In this study, *I. verticillata* leaves began to lose their green color and dry out at a later stage of drought stress (**Figure 1**). The relative leaf water content and SLW decreased with increasing drought stress and the duration of drought (**Figure 2**).The results of this study are consistent with those of tomato (*Solanum lycopersicum* L.) (Gaion et al., 2018), sand holly (*Ammopiptanthus mongolicus* (Maxim. ex Kom.) Cheng f.) (Huo, 2010), beech bean (*Tephrosia candida* DC.) (Chen et al., 2004), alfalfa (*Medicago sativa* L.) (Zhang, 2015) and other plants. The exogenous application of SPD alleviated the greening and drying of leaves and increased the RWC and SLW of leaves under drought stress. This suggests that SPD improves the drought resistance of *I. verticillata* seedlings. We discovered that leaves of the native species have leathery characteristics by comparing the leaf phenotypes and SLWs of the three different varieties under drought stress. It had a higher RWC, chlorophyll content, and Pn under drought stress than the other two *I. verticillata* varieties, indicating that it was more drought resistant than the two varieties.

### 4.2 Exogenous SPD appears to ameliorate drought stress by increasing photosynthesis in plants

Plant tolerance to water deficit is associated with an increase in chlorophyll content, as well as an increase in the relative leaf water content. The dehydration of leaves can damage the cell membrane system of plant leaves and inhibit the synthesis of chlorophyll (Duan et al., 2006). Many studies have shown that the chlorophyll and leaf nitrogen contents are progressively reduced under drought stress (Jeyaramraja et al., 2005). In this study, the chlorophyll content of *I. verticillata* leaves gradually decreased as the degree of drought stress deepened, particularly in the late stages of drought stress when the contents of both chlorophyll and leaf nitrogen decreased significantly (**Figure 3**), which is consistent with the findings in wheat (*Triticum aestivum* L.) (Yang et al., 2004). During the late stages of severe drought stress, Fv/Fm, Y(II), ETR, and qP were significantly higher in groups in which exogenous SPD had been applied than in the non-SPD treated groups, while no significant regular differences in NPQ and qN were observed. Drought stress directly affects the photosynthetic efficiency of plants. The decrease in nitrogen content impairs chlorophyll synthesis in the leaves, which results in a decrease in Fv/Fm, Y(II), ETR, and qP, and thus, subsequent damage to the plant’s photosynthetic system. In short, exogenous SPD can increase the photosynthetic efficiency of *I. verticillata* leaves under drought stress by increasing the chlorophyll content, Fv/Fm, Ti, Gs.

### 4.3 Exogenous SPD could alleviate drought stress by regulating peroxidation processes

Plants typically produce and remove ROS in a relatively dynamic balance. Under adverse conditions, ROS can significantly increase, causing membrane peroxidation, which is highly toxic to organisms. Plants primarily scavenge free radicals through enzyme systems, such as SOD, POD, and CAT, as well as antioxidant substances. Changes in the content of MDA provide an indication of the extent of membrane lipid peroxidation, which enables the indirect measurement of the extent of damage to the membrane system and the plant’s resistance to stress.

Membrane lipid peroxidation is the oxidation of phospholipid molecules, the structural backbone of cell membranes, to produce peroxides, which can damage the cell membrane. According to the theory of biological free radical damage, the production of large amounts of free radicals in plants causes membrane lipid peroxidation, which leads to disruption of the cell membrane system, interference with plant photosynthesis, and, in extreme cases, plant cell death (Khaerunnisa et al., 2020). MDA is one of the most important products of membrane lipid peroxidation; thus, an increase in the MDA content indicates membrane lipid peroxidation in plants (Idrees et al., 2011; Yang, 2018; Liu et al., 2021). This study reported that the exogenous application of polyamines (PAs) can reduce the stress-induced inhibition of growth, increase antioxidant enzyme activity, and reduce the accumulation of ROS (Tanou et al., 2014; Du et al., 2015). This study found that the MDA content of *I*. *verticillata* leaves increased with drought stress, most likely owing to the peroxidation of plant membrane lipids, a finding that is consistent with a similar study in tobacco (*Nicotiana tabacum* L.) (Han et al., 2022). Exogenously applied SPD significantly reduced the levels of MDA (**Figure 5A**). Simultaneously, the activities of SOD, CAT and POD increased and then decreased with time (**Figures 5B-D**) probably owing to the production of various antioxidants, such as antioxidant enzymes and lipid-soluble and water-soluble molecules, to scavenge ROS during the early stages of drought stress in plants to protect themselves from peroxidative damage (Dodd and Davies, 2010). In contrast, plants have a limited ability to regulate themselves, and as the duration of drought stress increases, the plant is damaged, which results in a decrease in the activity of antioxidant enzymes until the plant dies as discovered in a study of tomato (Luo and Polytechnic, 2018). In this study, the exogenous application of SPD reduced the SOD activity of *I. verticillata* to some extent, but increased the activities of POD and CAT. Thus, exogenous SPD could enhance the drought resistance of *I. verticillata* mainly by indirectly regulating the antioxidant system.

### 4.4 Exogenous SPD may alleviate osmoregulatory stress by lowering the levels of SS

Several studies have found that plants adapt to drought stress through other mechanisms, such as osmotic and plant hormone regulation (Guo et al., 2017; Mahmoudi et al., 2019). Under drought stress, the content of SS increases to reduce the intracellular water potential for more vigorous water uptake, which is consistent with the findings in this study (Zhang et al., 2020). Under drought stress, the content of SS increases to reduce the intracellular water potential (Zhang et al., 2020). The SS content of *I. verticillata* leaves was significantly reduced by the exogenous application of SPD (**Figure 6A**), implying that exogenous SPD could alleviate the osmotic regulation by reducing the SS content.

Many plant hormones, including IAA, ABA, and GA, have been implicated in the stress response in numerous scientific reports (Yin et al., 2009; Ji et al., 2011; Zhou et al., 2014; Kaundal et al., 2017). Abiotic stresses, such as drought, high salinity, and low temperature, induce the accumulation of ABA, which leads to increased tolerance, whereas inhibiting GA levels promotes tolerance to abiotic stresses, such as drought (Nir et al., 2017). IAA may also play a crucial role in the response of plants to drought stress (Du et al., 2012). In this study, the levels of IAA and GA gradually decreased with increasing drought and duration of drought stress, which is consistent with previous reports on cucumber (*Cucumis sativus* L.) (Zhang et al., 2009), maize (Li et al., 2018a; Li et al., 2018b) and centipedegrass (*Eremochloa ophiuroides* [Munro] Hack.) (Liu et al., 2017). Furthermore, the ABA content of *I. verticillata* leaves increased with time and drought, as did their drought resistance, which was consistent with the findings. However, the application of exogenous SPD had an inhibitory effect on the increase in ABA in *I. verticillata* leaves, indirectly indicating that exogenous SPD was not conducive for plants to manage drought stress through ABA in *I. verticillata*. This contrasts with the findings of Capell et al. (2004) (Capell et al., 2004), who showed that the overexpression and biosynthesis of spermine or the exogenous application of spermidine increased tolerance to drought stress (Zierer et al., 2016). These findings indicate that this is owing to the exogenous SPD that reduced the activity of *I. verticillata* SOD to some extent, a reference that merits further verification.

## 5 Conclusions

The phenotypic, physiological, and biochemical changes of *I. verticillata* (L.) A. Gray and its two representative varieties were studied under drought stress conditions. Moreover, the effect of exogenous SPD on the drought stress response of *I. verticillata* was investigated. The exogenous application of SPD could alleviate drought stress in *I. verticillata* by increasing the photosynthetic and antioxidant abilities, decreasing the peroxide content, and regulating the endogenous hormone content according to the comprehensive approach. The results also revealed that ‘Oosterwijk’ and ‘Jim Dandy’ are less drought resistant, whereas the native species is more drought resistant, providing important insights into the drought resistance of *I. verticillata*. As a result, *I. verticillata* (L.) A. Gray could be a candidate variety to have the potential to be cultivated in arid and semiarid areas.

## Data availability statement

The original contributions presented in the study are included in the article/**Supplementary Material**. Further inquiries can be directed to the corresponding author.

## Author contributions

HY and BZ conceived and designed the concept of manuscript. WW, XX, YG, SQ, SF, JM, JW, DM, GW, and YY performed the experiments. XX and WW analyzed the data. XX, FA, HY, and AS did the formal analysis. WW, XX, YG, SQ, SF, JM, JW, DM, GW, and YY investigated the data. HY and BZ provided resources. WW and XX curated the data. WW and XX drafted the manuscript. FA, HY, XX, and AS revised and finalized the manuscript. XX, YG, WW, XW, DY, and YH visualized the manuscript. All authors contributed to the article and approved the submitted version.
