# Supplementary material for: Exogenous spermidine improved drought tolerance in Ilex verticillata seedlings
Source: Front Plant Sci. 2023 Jan 20;14:1065208. doi: 10.3389/fpls.2023.1065208 (PMC9895825; doi:10.3389/fpls.2023.1065208)
Supplement: Supplementary file 1 [file Presentation_1.pdf]

Supplementary Material

1 Supplementary Figures

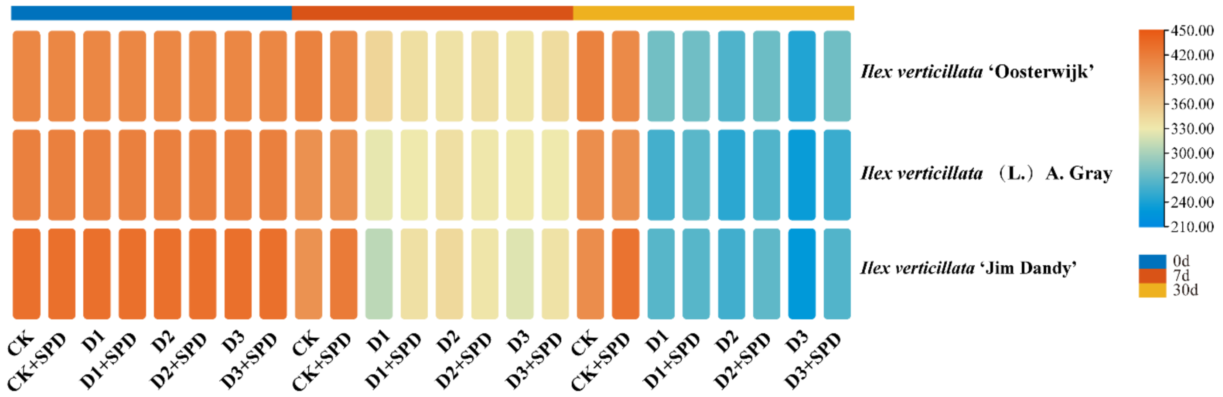

Supplementary Figure 1. The changes of intercellular CO_2 concentration in *I. verticillata* leaves under drought and spermidine treatment. Data were shown in a heatmap, where orange indicates high values and blue indicates low values. CK, control; CK+SPD, control with spermidine; D1, mild drought; D1+SPD, mild drought with spermidine; D2, moderate drought; D2+SPD, moderate drought with spermidine; D3, severe drought; D3+SPD, severe drought with spermidine.

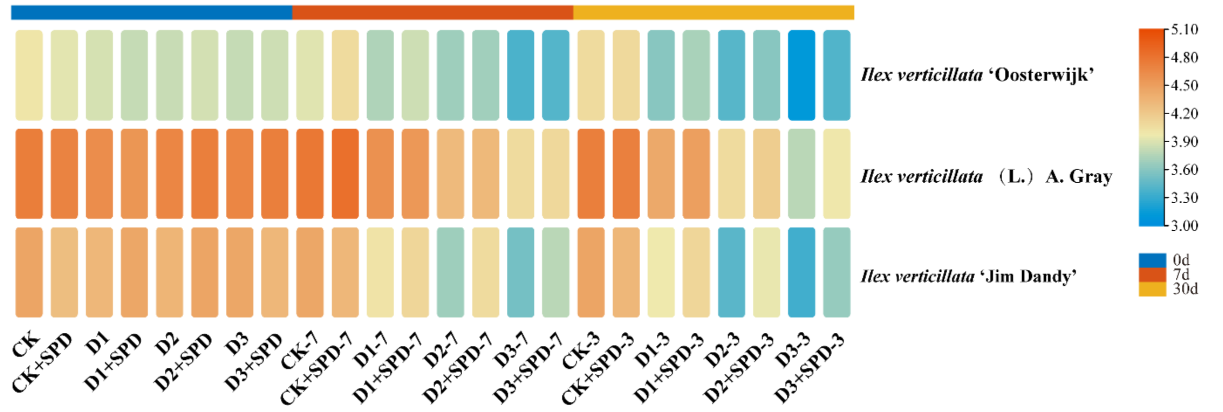

Supplementary Figure 2. The changes of nitrogen content in *I. verticillata* leaves under drought and spermidine treatment. Data were shown in a heatmap, where orange indicates high values and blue indicates low values. CK, control; CK+SPD, control with spermidine; D1, mild drought; D1+SPD,

mild drought with spermidine; D2, moderate drought; D2+SPD, moderate drought with spermidine; D3, severe drought; D3+SPD, severe drought with spermidine.

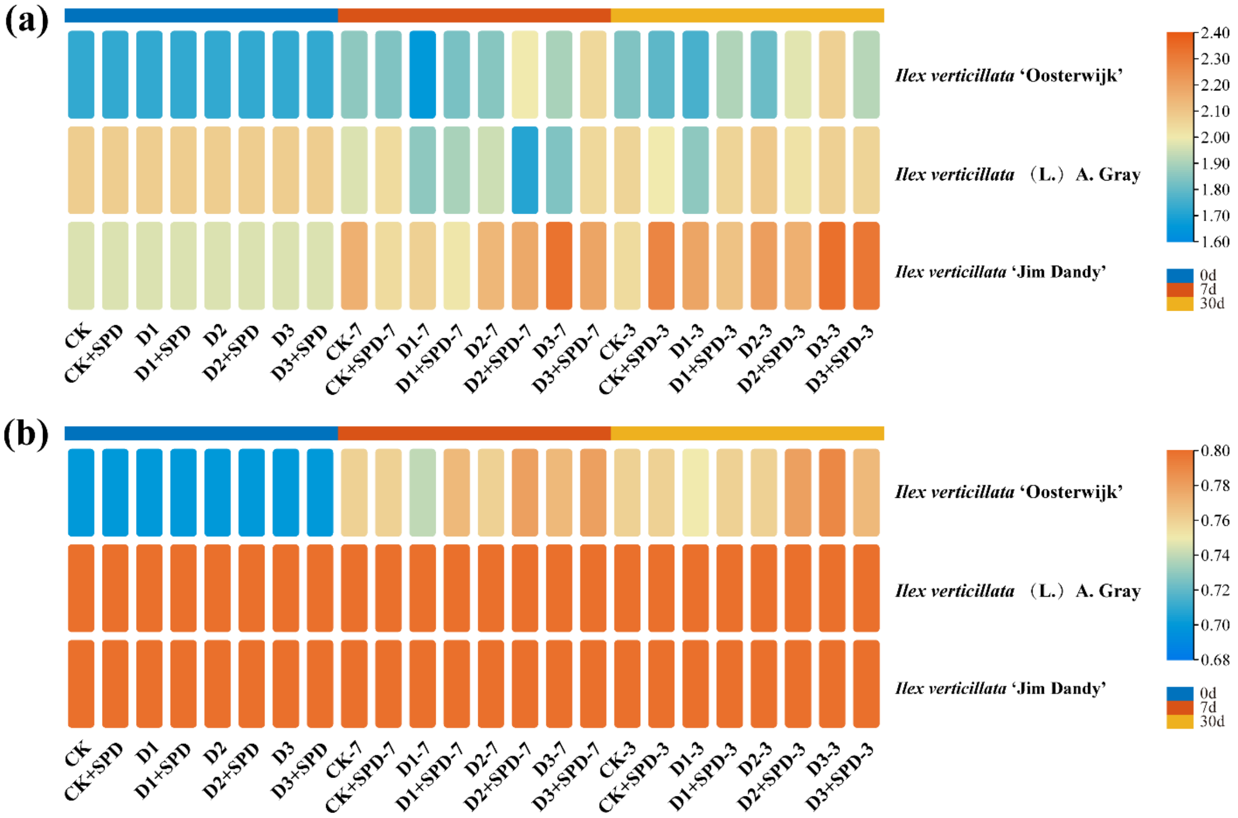

Supplementary Figure 3. The changes of NPQ and qN in *I. verticillata* leaves under drought and spermidine treatment. (a) NPQ; (b) qN. Data were shown in the heatmaps, where orange indicates high values and blue indicates low values. CK, control; CK+SPD, control with spermidine; D1, mild drought; D1+SPD, mild drought with spermidine; D2, moderate drought; D2+SPD, moderate drought with spermidine; D3, severe drought; D3+SPD, severe drought with spermidine. Data on 0d, 7d and 30d were conducted for one-way analysis of variance (ANOVA, $P < 0.05$, n = 3), respectively. Multiple comparisons were conducted using Duncan's method. Different lowercase letters after the data on the same day indicate significant difference between different treatments, while the same lowercase letters indicate non-significant difference.
